# Current and novel infusion therapies for patients with Parkinson's disease

**DOI:** 10.1007/s00702-023-02693-8

**Published:** 2023-09-06

**Authors:** Angelo Antonini, Valentina D’Onofrio, Andrea Guerra

**Affiliations:** 1 Parkinson and Movement Disorders Unit, Centre for Rare Neurological Diseases (ERN-RND), Department of Neuroscience, University of Padua, Via Giustiniani 3, 35121 Padua, Italy; 2 Padova Neuroscience Center (PNC), University of Padua, Padua, Italy

**Keywords:** Parkinson’s disease, Device-aided therapies, Infusion therapies, Apomorphine, Levodopa/carbidopa intestinal gel, Subcutaneous levodopa

## Abstract

Advanced Parkinson’s disease is characterized by periods of poor mobility, dyskinesia and progressive decline in functional independence of the affected person despite the manipulation of levodopa doses and the introduction of supplemental therapies such as catechol-O-methyl transferase inhibitors, monoamine oxidase-B inhibitors and dopamine agonists. The implementation of drug delivery systems allows to bypass problems related to irregular and often unpredictable intestinal absorption of oral levodopa, which significantly affects its bioavailability and contributes to the development and persistence of motor complications. Subcutaneous apomorphine and levodopa/carbidopa jejunal infusion systems have been available for many years and their efficacy is confirmed by randomized studies and long-term experience in many centers worldwide. Recently, a new formulation of levodopa/carbidopa infusion gel that includes the catechol-O-methyl transferase inhibitor Entacapone has been introduced to the market. The use of entacapone allows to reduce total daily dose of administered levodopa. Two different soluble formulations of levodopa/carbidopa (ND0612 and ABBV-951) have completed clinical development, and both can ensure subcutaneous delivery by a portable pump infusion system. ABBV-951 uses a foslevodopa/foscarbidopa formulation, both prodrugs to improve absorption and tolerability. Both systems provide effective improvement of motor complications and are likely to expand the therapeutic options in advanced patients. Future efforts should focus on the earlier detection of patients who are candidates for device-aided therapies, increasing appropriate referral and broadening the availability of these treatments globally.

## Introduction

Parkinson’s disease (PD) is a progressive neurodegenerative disorder characterized by loss of nigrostriatal dopamine neurons and its incidence is increasing fast globally (Kalia and Lang 2015; Poewe et al. 2017). In the first years of disease and after the onset of motor manifestations, spared dopaminergic neurons preserve the capacity to convert, store and release dopamine in a relatively tonic manner, so that dopamine concentration is maintained at physiological levels in the synaptic cleft despite the short half-life of oral levodopa (Rascol et al. 2003). When the loss of nigrostriatal nerve terminals becomes severe, the neurotransmitter storage capacity diminishes and dopamine levels in the central nervous system are progressively dependent on the pharmacokinetics of exogenous levodopa (Di Monte et al. 2000; Nyholm 2007; Bastide et al. 2015). Erratic gastric emptying and variable jejunal absorption eventually contribute to oscillating levodopa plasma levels and in turn to variable extra-synaptic dopamine concentration (Bestetti et al. 2017; Chaudhuri et al. 2015). Pulsatile stimulation of striatal dopamine receptors and molecular and plastic changes in the basal ganglia, thalamus and cerebral cortex are considered key elements in the development of motor complications, such as fluctuations of clinical status, delayed-ON, wearing-OFF and dyskinesia (Antonini et al. 2011; Bastide et al. 2015; Picconi et al. 2018; Fabbrini and Guerra 2021). As a result, the time spent in a good mobility (ON time) decreases and the time with inadequate clinical efficacy (OFF time) increases along with disease progression (Olanow et al. 2006; Chaudhuri et al. 2013). Several therapeutic strategies including dopamine agonists (DA), catechol-O-methyltransferase (COMT) and monoamine oxidase-B (MAO-B) inhibitors are available, but they cannot completely abolish complications which in most patients become progressively impactful on quality of life (QoL) and functional independence.

Advanced PD (APD) is defined as a condition where periods of poor mobility with or without dyskinesia are present and have an impact on functional independence of the affected person (Antonini et al. 2018b, a). In this condition adjunctive treatment options to levodopa, including dopamine agonists (DA), catechol-O-methyltransferase (COMT) inhibitors and monoamine oxidase-B (MAO-B) inhibitors should be considered, as well as devise aided therapies (Antonini et al. 2018b, a; Armstrong and Okun 2020).

**Fig. 1:**
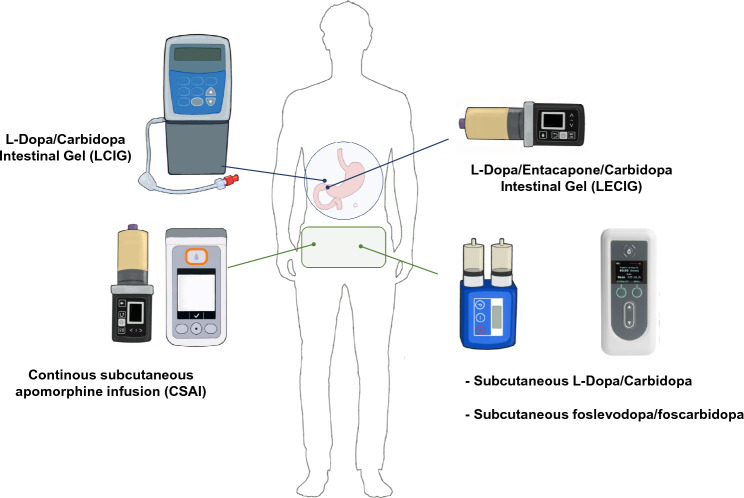
Overview of currently available and upcoming infusion systems in Parkinson’s disease

The rationale for infusion therapies is to achieve continuous dopaminergic stimulation. Constant plasma levels of levodopa or of the DA apomorphine, minimize adverse events from polytherapy and improve treatment adherence, reduce dopaminergic hypersensitivity and minimize cardinal mechanisms underlying the pathophysiology of motor complications (Rascol et al. 2003; Olanow et al. 2020; Fabbrini and Guerra 2021; Kolmančič et al. 2022; Van Laar et al. 2023). Infusion therapies bypass problems related to irregular and often unpredictable intestinal absorption of oral levodopa, significantly affecting its bioavailability (Bestetti et al. 2017; Chaudhuri et al. 2015). Infusion therapies can also provide greater improvement than oral medications in advanced PD (APD) patients with poor symptoms control (Prakash and Simuni 2020; Antonini et al. 2022; Dijk et al. 2020). In line with this evidence, recent European Academy of Neurology/Movement Disorder Society (EAN-MDS) guidelines on the treatment of PD suggested them as a valid alternative to oral drugs in patients with APD and symptom fluctuations (Deuschl et al. 2022). Deep brain stimulation (DBS) is another effective therapy in patients with APD (Limousin and Foltynie 2019; Mahlknecht et al. 2022), but discussing surgical treatments goes beyond the purposes of this review. To date, two types of infusion therapies are available on the market (Fig. 1): continuous subcutaneous apomorphine infusion (CSAI) (Stibe et al. 1987; Poewe et al. 1988; Antonini and Jenner 2018) and intrajejunal infusion of levodopa/carbidopa intestinal gel (LCIG) (Nilsson et al. 1998; Tsunemi et al. 2021).

In this work, we will briefly review previous studies with LCIG and CSAI in APD patients. We acknowledge that other recent reviews on the topic exist (Prakash and Simuni 2020; Van Laar et al. 2023). We will focus on novel approaches and drug formulations for infusion pump devices, which are currently in the process of registration and market authorization (Fig. 1). Finally, we will provide critical comments on the possible future of infusion treatments for PD, also considering the significant technological advances achieved in device-aided therapies over the last decade.

## Current infusion therapies available for patients with Parkinson’s disease

### Continuous subcutaneous apomorphine infusion (CSAI)

**Fig. 2:**
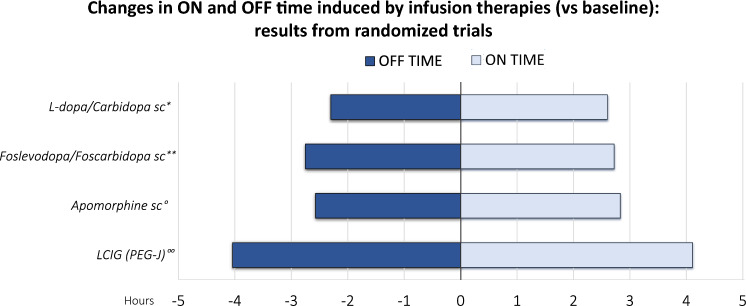
Changes in the duration of ON and OFF time (in hours) from different infusion therapies with respect to the baseline scores. *Poewe et al. 2021; **Soileau et al. 2022; °Katzenschlager et al. 2018; °°Olanow et al. 2014. *This study was not randomized and open-label but results were confirmed by the BouNDless study (in press)

CSAI was the first infusion therapy available in Europe and its introduction to the market dates back to the early 1990s (Stibe et al. 1987; Poewe et al. 1988). Apomorphine hydrochloride is a DA with similar efficacy to levodopa as it acts on both D1 and D2 receptors, but has faster and shorter-lasting effects, characteristics that made it useful as ‘rescue therapy’ for sudden OFF phenomena (Kempster et al. 1990; Merello et al. 1997; Jenner and Katzenschlager 2016, p. 201; Antonini and Jenner 2018). However, due to its poor bioavailability, subcutaneous continuous administration is required to guarantee constant antiparkinsonian effects. CSAI can reduce the daily OFF time and increase the ON time (36–80% depending on the study) (Carbone et al. 2019; Prakash and Simuni 2020; Van Laar et al. 2023). If tolerated, CSAI dosage can be increased leading to discontinuation of oral therapies with levodopa and/or oral DA and use of CSAI as their sole dopaminergic therapy (Jenner and Katzenschlager 2016). Efficacy relies on numerous open-label studies conducted over the past years (Borgemeester et al. 2016; Sesar et al. 2017; Van Laar et al. 2023), but there is only one international multicentre, randomized, double-blind, placebo-controlled trial (Katzenschlager et al. 2018) available (Fig. 2). The TOLEDO study showed a > 2-h reduction in daily OFF time after 12 weeks of treatment in > 50% of patients, with a mean reduction in OFF time of 1.89 h and an increase in the ON time without problematic dyskinesia by 1.97 h compared to placebo. The open-label phase extension confirmed a significant reduction in daily OFF time and improvement in ON time without troublesome dyskinesia, which was sustained for up to 64 weeks (Katzenschlager et al. 2021). A significant improvement in dyskinesia duration and severity (31–57% reduction) has also been reported in various studies. However, the sample size of these studies was small, the design was open-label, and the apparent antidyskinetic effect was related to the decrease in levodopa equivalent daily dose (LEDD) (Stocchi et al. 2001; García Ruiz et al. 2008). Accordingly, recent EAN-MDS guidelines and meta-analyses recognized that there is insufficient controlled data to establish whether CSAI is an effective treatment in APD patients with dyskinesia (Deuschl et al. 2022; Antonini et al. 2022). CSAI medium-to-long-term therapy was paralleled by amelioration in several non-motor domains, including sleep, restlessness, urinary dysfunction, gastrointestinal symptoms, fatigue and apathy (Todorova and Ray Chaudhuri 2013; Martinez-Martin et al. 2015; Rosa-Grilo et al. 2016). The effect of CSAI on QoL is uncertain. Although QoL did not improve neither in the TOLEDO study nor in its open-label extension phase (Katzenschlager et al. 2018, 2021), the results of real-world and observational studies demonstrated QoL amelioration after 6 months of chronic treatment (Martinez-Martin et al. 2015; Drapier et al. 2016; Houvenaghel et al. 2018). Importantly, CSAI therapy may lead to possible neuropsychiatric complications, such as visual hallucinations and cognitive deterioration (Martinez-Martin et al. 2015; Sesar et al. 2017; Olivola et al. 2019). However, such complications are often observed in patients with cognitive deficits and are usually not severe (Hughes et al. 1993; Pietz et al. 1998). In addition, nausea, somnolence and hypotension may develop at the beginning of therapy. The most common side effect during CSAI is, however, the occurrence/development of nodular skin reactions at the infusion site (54.8% of patients enrolled in the TOLEDO study from the baseline of the double-blind phase to the end of the open-label phase) (Katzenschlager et al. 2018, 2021). Although CSAI-induced skin reactions are mild or moderate in most patients and can be partly prevented by following daily management recommendations, in rare cases, serious local adverse events can cause treatment discontinuation (Pietz et al. 1998; Jenner and Katzenschlager 2016).

### Levodopa/carbidopa intestinal gel infusion (LCIG)

LCIG therapy consists of a gel suspension of levodopa/carbidopa monohydrate (4:1 ratio) in a water solution of carboxymethyl cellulose into the patient’s proximal small intestine through a portable pump and a tube placed via percutaneous endoscopic gastro-jejunostomy (PEG-J) (Fig. 1). Importantly, this approach allows to avoid pulsatile stimulation due to erratic gastric emptying (Bestetti et al. 2017). Treatment is usually administered during the patients’ awake period (≈16 h), but it may be administered for up to 24 h if medically justified (i.e., overnight wearing off symptoms, severe night-time pain or delayed morning latency) (Prakash and Simuni 2020; Thakkar et al. 2021; Antonini et al. 2021; Tsunemi et al. 2021).

There is one multicentre double-blind, randomized, placebo-controlled clinical trial demonstrating the efficacy of LCIG over standard oral therapy where APD patients treated with immediate-release oral levodopa/carbidopa vs placebo intestinal gel infusion to a group taking LCIG infusion and oral placebo (Olanow et al. 2014). The results showed a reduction in daily OFF time of 1.91 h and an improvement in ON time without problematic dyskinesia of 1.86 h after 12 weeks of treatment in the LCIG group compared with the placebo intestinal gel group (Fig. 2). Subsequent studies have shown that these benefits are not only maintained in the long-term, but a further reduction in total OFF time is also observed in the long-term (Slevin et al. 2015; Fernandez et al. 2015, 2018; Antonini et al. 2017). Concerning the effects on dyskinesia, some studies have documented a reduction in the ON time with both short-term and long-term improvement of troublesome dyskinesia after LCIG therapy (Antonini et al. 2016; Lopiano et al. 2019). Furthermore, when examining in detail the subgroup of patients with severe and persistent dyskinesia at baseline, the data showed significant improvement in several features of the dyskinesia, including duration, severity and associated pain (Poewe et al. 2019). Investigating the effectiveness of LCIG on dyskinesia was the primary aim of a recent open-label, multicentre, 12-week, interventional study (DYSCOVER study) (Freire-Alvarez et al. 2021). The results showed a significant reduction in the Unified Dyskinesia Rating Scale (UDysRS) score (− 15.05 ± 3.20) and improvement in dyskinesia-related pain and early morning dystonia in the LCIG group compared with the optimized medical treatment (OMT) group after 12 weeks. Multicentre clinical trials have also documented the efficacy of LCIG therapy on non-motor symptoms of PD including sleep, fatigue, mood, and cardiovascular, cognitive, gastrointestinal, urinary, sexual, perceptual/hallucinations symptoms (Antonini et al. 2015, 2017; Lopiano et al. 2019; Fasano et al. 2021; Standaert et al. 2021). Interestingly, in a prospective, observational, multicentre study with an open-label design comparing CSAI and LCIG (EUROINF study), both treatments determined a marked and comparable improvement in motor symptoms but the overall effect was observed more frequently in patients on LCIG (Martinez-Martin et al. 2015). Moreover, these patients also had benefit on gastrointestinal, urinary, sexual and sleep disturbances and fatigue. The positive effect on the ON and OFF time, dyskinesia as well as non-motor symptoms is also reflected by the significant improvement of QoL and activities of daily living (ADL) using LCIG with respect to OMT (Nyholm et al. 2005; Olanow et al. 2014; Freire-Alvarez et al. 2021; Rus et al. 2022). Confirming the results of previous clinical trials, a large real-world study (DUOGLOBE) recently showed that the LCIG therapy is clearly effective in ameliorating motor (OFF time and dyskinesia) and non-motor symptoms and QoL in APD patients, and determined a significant reduction in the caregiver burden after 12 months of therapy (Standaert et al. 2021). Notably, data from long-term studies showed that LCIG is increasingly used as a monotherapy. Namely, the amount of APD patients treated with LCIG monotherapy doubled in a year (from ≈15 to ≈30%) (Fasano et al. 2021). Also, the amount of improvement in motor and non-motor symptoms and ADL was comparable between patients receiving LCIG as monotherapy and polytherapy (Antonini et al. 2017; Poewe et al. 2019).

LCIG therapy has an acceptable long-term safety profile and a low rate (≈25%) of withdrawal (Garrì et al. 2022). However, LCIG has a high rate of adverse effects (55–90% of patients), most of which are mild or moderate in severity (Fernandez et al. 2015, 2018; Lang et al. 2016; Antonini et al. 2017; Lopiano et al. 2019; Standaert et al. 2021). The most common ones relate to the surgical procedure, ranging from abdominal pain and surgical wound infection to severe, but rare complications, such as peritonitis or pneumonia, which generally occur within the first four weeks after PEG-J placement (Lang et al. 2016; Antonini et al. 2017; Standaert et al. 2021). In the longer term, intestinal malabsorption symptoms and, in 5–10% of patients, a polyneuropathy may occur, which is possibly related to a complex interplay between peripheral neurodegenerative processes, the effects of levodopa neurotoxic metabolites and vitamin B12 and B6 deficiency (Müller et al. 2013; Mancini et al. 2014; Romagnolo et al. 2019). In real-life experience, APD patients may report some discomfort and practical difficulties caused by the device dimension and weight (e.g., when dressing or walking long distances), minor management duties (e.g., daily cleaning of the tube or battery changes) and travelling issues due to the need of cassettes containing the drug to be refrigerated.

## New infusion therapies for patients with APD

### Levodopa/entacapone/carbidopa intestinal gel (LECIG)

LECIG is a novel infusion treatment consisting of a triple combination of levodopa, carbidopa and entacapone (Fig. 1). Similar to LCIG, LECIG is continuously delivered into the duodenum/jejunum through a PEG-J and a portable pump. The innovation is related to the addition of the COMT inhibitor entacapone to the levodopa/carbidopa gel, which increases the levodopa plasma concentration by blocks the second-largest degradation pathway and reduces levodopa conversion to 3-O-methyldopa, thereby increasing the its plasma concentration and reducing the daily dose (Müller 2010; Nyholm and Jost 2022; Fabbri et al. 2022). The smaller infusion volume allows also the use of smaller and lighter infusion pumps (Nyholm and Jost 2022; Jost 2023). Although a small pump is widely perceived as a relevant advantage in terms of patients’ comfort and device portability, smaller or highly computerized interfaces might be challenging for some patients/caregivers (Öthman et al. 2021; Klarich et al. 2022; Jost 2023). Efficacy data are limited to a randomized, open-label, crossover, pharmacokinetic study conducted on 11 APD patients where the systemic levodopa exposure of LECIG was compared to LCIG. The results showed comparable exposure between LCIG and LECIG with a 20% reduction in infusion dose vs LCIG (Senek et al. 2017). Importantly, treatment response scale (TRS) scores were similar between treatments. The suitable dose conversion from LCIG to LECIG treatment was better characterized in a later study (Senek et al. 2020). The continuous maintenance dose of LECIG should be reduced by ≈35% on average to achieve similar drug exposure as with LCIG, and interestingly this result was not influenced by the patient’s COMT genotype. The first clinical experience with LECIG was conducted in a single-centre observational study on 24 APD patients who were treated for > 10 months (Öthman et al. 2021). As expected, a high proportion of patients who were not previously treated by levodopa infusion reported a significant improvement in ADL, QoL and motor symptoms. Confirming previous pharmacokinetic studies, patients who switched from LCIG to LECIG perceived that the clinical effect of infusion therapy did not change (note that the infusion rate was reduced by 24% compared to their previous LCIG dose in this study) (Öthman et al. 2021). To date, LECIG is approved only in some European countries. A large multicentre international, prospective, non-interventional, observational study is ongoing to collect data on the long-term (24 months) effectiveness and safety of LECIG in patients with APD in routine clinical practice (ELEGANCE study, NCT05043103) (Jost 2023). The planned total number of patients to be recruited is ≈300 (APD patients with no history of infusion therapy or patients who switch from another infusion therapy) and the estimated study completion is in July 2025. The primary endpoints of the study are the LECIG effects on motor symptoms (daily OFF time and motor aspects of experiences of daily living), daily levodopa dose, the use of other PD medications, Clinical and Patient Global Impression and the satisfaction with LECIG treatment (for more details on the study design see Jost 2023). There is currently limited evidence on the safety profile of LECIG. The only clinical study performed to date reported that side effects leading to treatment discontinuation were diarrhoea and hallucinations. There were also one cardiac arrest and one patient’s death for COVID-19, which were unrelated to LECIG treatment (Öthman et al. 2021).

### Levodopa/carbidopa continuous subcutaneous infusion (LC-CSCI)

LC-CSCI therapy has been developed in recent years and consists of a soluble formulation of levodopa/carbidopa delivered subcutaneously by a portable pump infusion similar to that used for CSAI. Two compounds are currently in an advanced phase of investigation and are close to be introduced to global markets: ND0612 (Olanow et al. 2021; Poewe et al. 2021) and ABBV-951 (Rosebraugh et al. 2021, 2022; Soileau et al. 2022) (Fig. 1).

ND0612 is a drug-device combination consisting of a sterile solution of levodopa/carbidopa continuously delivered via a dedicated subcutaneous pump (Olanow et al. 2021). In a series of clinical pharmacokinetic phase 1 and 2 studies, ND0612 infusion has been shown to lead to stable and therapeutic plasma concentrations of levodopa (LeWitt et al. 2022). In particular, in a recent randomized placebo-controlled study, patients treated with ND0612 as add-on therapy to their OMT showed reduced variability and the disappearance of deep troughs in levodopa plasma levels compared to placebo. (Giladi et al. 2021). Feasibility, safety and preliminary evidence of the efficacy of ND0612 come from a 28-day open-label study (NCT02577523) (Olanow et al. 2021), where 33 APD patients were randomized in two groups based on the ND0612 dosing regimen (24-h infusion vs. 14-h ‘waking-day’ infusion). In the overall population, ND0612 decreased the OFF time by ≈2 h and the ON time with moderate-to-severe dyskinesia by ≈1.2 h and increased the ON time without troublesome dyskinesia by ≈3.3 h. However, patients in the 24-h infusion group demonstrated a greater reduction in the OFF time when compared to patients in the 14-h group (− 2.8 vs. − 1.3 h on average). In both groups, adverse events were frequent (at least one adverse event in more than 75% of patients), and the vast majority were related to infusion site reactions (nodule, bruising, erythema, haemorrhage, hematoma, pain, oedema and pruritis) (Olanow et al. 2021). However, an international, multicentre, 102-month, open-label study is ongoing with the primary aim of evaluating the long-term safety and tolerability of ND0612 (BeyoND study, NCT02726386). The estimated study completion date is February 2027, but preliminary data have been recently published (Poewe et al. 2021). One hundred and twenty out of 214 enrolled patients (56%) completed 12 months of treatment and 85.5% of patients reported at least one adverse event (Fig. 2). Adverse events were mainly mild-to-moderate in severity and led to study discontinuation in 17.3% of cases. Confirming previous evidence (Olanow et al. 2021), the most common adverse events were infusion site reactions (skin nodules, hematoma or, more rarely, infections), followed by the worsening of dyskinesia (Poewe et al. 2021). These results indicate that LC-CSCI with ND0612 is safe, but infusion site reactions related to the subcutaneous route of administration are common and may lead to treatment discontinuation. Recently, results from the BouNDless study (NCT04006210) were announced. BouNDless is a phase 3 randomized, active-controlled, double-blind, double-dummy trial designed to establish the efficacy, safety and tolerability of ND0612 in comparison to oral immediate-release levodopa/carbidopa in patients with Parkinson’s disease experiencing motor fluctuations. Following two sequential open-label periods to optimize oral LD/CD and ND0612, patients were randomized to either ND0612 or oral LD/CD for a 12-week DBDD period. Treatment with ND0612 demonstrated superiority over oral LD/CD, with a statistically significant difference (*p* < 0.0001) of 1.72 h in “Good ON” time. The trial also demonstrated positive and clinically meaningful results for the key secondary endpoint of “OFF” time (*p* < 0.0001) and other secondary endpoints including the MDS-Unified Parkinson's Disease Rating Scale Part II score (MDS-UPDRS motor experiences of daily living sub-score) (*p* < 0.0001); the Patient Global Impression of Change (PGIC) (*p* < 0.0001); and the Clinical Global Impression of Improvement (CGI-I) (*p* < 0.0001).

ABBV-951 is a new soluble formulation of foslevodopa/foscarbidopa, both levodopa and carbidopa prodrugs, which can be delivered subcutaneously for up to 24 h/day. After administration, foslevodopa/foscarbidopa is quickly converted to levodopa/carbidopa by alkaline phosphatases, reaching and maintaining therapeutic steady-state plasma levels in a very short time (Rosebraugh et al. 2021, 2022; Soileau et al. 2022; Van Laar et al. 2023). In a first study on preclinical pharmacokinetic and phase 1 data in healthy volunteers, foslevodopa/foscarbidopa demonstrated high water solubility and excellent chemical stability near physiological pH, with stable levodopa pharmacokinetic profile for ≤ 72 h and good tolerability (Rosebraugh et al. 2021). A following phase 1, open-label, randomized, crossover study conducted in 25 healthy subjects compared levodopa pharmacokinetics from 24-h foslevodopa/foscarbidopa subcutaneous infusion and 16-h LCIG infusion followed by night-time oral levodopa/carbidopa dosing (Rosebraugh et al. 2022). Levodopa exposure following 24 h foslevodopa/foscarbidopa 700/35 mg was comparable to LCIG 350/87.5 mg infused over 16 h and followed by two 100/25 mg levodopa/carbidopa oral doses at 6 and 9 P.M. Moreover, the magnitude of levodopa plasma fluctuations in the first 16 h of infusion was comparably low in both treatments, suggesting that foslevodopa/foscarbidopa subcutaneous infusion maintains levodopa exposure within a narrow therapeutic window like LCIG (Rosebraugh et al. 2022). The pivotal study on the efficacy and safety of foslevodopa/foscarbidopa CSCI has been recently published (Soileau et al. 2022). In this multicentre, double-blind, double-dummy, active-controlled, phase 3 trial (NCT04380142), 141 patients with APD inadequately controlled by the OMT were randomized to 24-h foslevodopa/foscarbidopa CSCI and oral placebo or CSCI placebo solution and oral immediate-release levodopa/carbidopa (Soileau et al. 2022). After 12 weeks of treatment, patients in the foslevodopa/foscarbidopa CSCI group showed a significantly greater decrease in OFF time (mean difference of − 1.79 h) and an increase in ON time without troublesome dyskinesia (mean difference of 1.75 h) compared to the other group (Fig. 2). Most non-serious and mild-to-moderate adverse events in the foslevodopa/foscarbidopa CSCI group were infusion site reactions (72% of patients), while serious events which led to premature study drug discontinuation occurred in 22% of cases (Soileau et al. 2022). More recent, unpublished studies based on post hoc analyses of data from the pivotal study evaluated the effectiveness of foslevodopa/foscarbidopa CSCI on several additional motor and non-motor symptoms. Patients treated with 24 h/day foslevodopa/foscarbidopa CSCI woke up in good ON time more rapidly and frequently than patients taking oral levodopa/carbidopa, achieved a greater improvement and stability of their good ON time throughout the day and experienced fewer motor fluctuations (Pahwa et al. 2022). A phase 3, 52-week, open-label, single-arm, multicentre study evaluating the safety, tolerability and efficacy of foslevodopa/foscarbidopa delivered 24 h/day is ongoing (NCT03781167). Preliminary analyses on the patterns of concomitant medication use and LEDD during foslevodopa/foscarbidopa CSCI treatment showed that the proportion of patients using ≥ 2 classes of concomitant medications decreased at week 52. Also, more than 25% of patients were treated with foslevodopa/foscarbidopa monotherapy and LEDD remained stable throughout the treatment period (Santos Garcia et al. 2022). Finally, when examining the possible impact of age and disease duration on foslevodopa/foscarbidopa efficacy and safety, the findings from 6-month interim analysis demonstrated that these variables did not influence the magnitude of improvement in the OFF time and the overall benefit/risk profile. However, older patients showed higher rates of severe and serious treatment-emergent adverse events (Isaacson et al. 2022). The third interim analysis of the study has also shown that patients treated with 24 h/day foslevodopa/foscarbidopa had a significant improvement in QoL, as measured by the Parkinson’s Disease Questionnaire-39 item (PDQ-39) summary index total score and specific domains, including activities of daily living, mobility, bodily discomfort and stigma. The improvements were noted early after treatment initiation and were generally sustained throughout the 52-week treatment period (Gandor et al. 2023). Furthermore, the preliminary analysis on secondary outcome measures of the NCT03781167 study demonstrated that patients treated with 24 h/day foslevodopa/foscarbidopa for at least 26 weeks had a significant improvement in sleep quality. This effect positively correlated with the amelioration of OFF time, motor experiences of daily living and QoL, supporting the benefits of 24-h/day therapy in APD (Chaudhuri et al. 2022). When considering the data from NCT04380142 and NCT03781167 study, a post hoc analysis that evaluated falls, posture and freezing of gait found that these specific symptoms improved after foslevodopa/foscarbidopa and the amount of improvement was positively associated with patient QoL (Odin et al. 2023).

## Conclusions

Research in PD has increasingly focused on the development of new and effective biological therapies for disease modification (Antonini et al. 2020). However, PD patients will still require symptomatic treatments for the years to come, especially when they reach an advanced stage. Furthermore, symptomatic treatment represents the most reliable and feasible approach to improving patient QoL and overall well-being, regardless of underlying disease mechanisms. The impact of new infusion systems is, therefore, expected to be very important. In our opinion, it is conceivable that subcutaneous levodopa infusion will broaden the current use for these treatments not only to APD but also to patients in the early stages of fluctuations shortly after the introduction of dopamine replacement therapy. Subcutaneous levodopa delivery and improvement in formulations and pump systems may allow its implementation in a larger number of subjects compared to the jejunal systems. This will also benefit apomorphine which has been underused now for many years. Even if patients may not be able to tolerate subcutaneous delivery for many years, they will be more likely to consider jejunal infusion or deep brain stimulation which may extend clinical benefit and ensure adequate functional independence in activities of daily living. Finally, future research into novel drug delivery methods, such as nanotechnology-based carriers or implantable devices, could offer more efficient and targeted ways to continuously administer medications.

Limitations will still be related to the lack of direct comparative efficacy and safety studies among advanced treatments and the difficulty of identifying specific patients’ profiles.

Infusion therapies might also become more integrated with remote monitoring technologies and telemedicine, allowing healthcare providers to track patient responses, adjust treatments and offer support from a distance (Guerra et al. 2023).

Along with the progressive increase in the number of options, the selection of more appropriate device-aided therapy for APD will be more complex, it will involve multiple specialties as well as the carers and affected ones. A shared decision approach is, therefore, essential. While treatment decisions need to be individualized, the choice of device-aided therapies can be guided by some general principles based on the patient’s age, cognitive and behavioral status, dyskinesia and frailty.

Understanding the comparative benefits of each treatment provides additional information that can help patients, caregivers and providers in the selection of the most appropriate therapy to ensure optimal symptom control and improved QoL (Martinez-Martin et al. 2023). Future efforts should focus on the earlier detection of patients who are candidates for device-aided therapy, increasing appropriate referral and broadening the availability of these therapies globally including the potential to increase access to these treatments in the developing world.
